# Synthesis of Monodisperse Mesoporous Carbon Spheres/EPDM Rubber Composites and Their Enhancement Mechanical Properties

**DOI:** 10.3390/polym16030355

**Published:** 2024-01-28

**Authors:** Tong Zheng, Wenjing Jia, Hongjie Meng, Jiajie Li, Xundao Liu

**Affiliations:** 1 School of Material Science and Engineering, University of Jinan, Jinan 250022, China; zhengtong974@163.com (T.Z.);; 2 Shanghai Key Lab of Electrical Insulation and Thermal Aging, School of Chemistry and Chemical Engineering, Center of Hydrogen Science, Shanghai Jiao Tong University, Shanghai 200240, China

**Keywords:** monodisperse mesoporous carbon spheres, ethylene propylene diene monomer, composites, mechanical properties, computer simulation

## Abstract

Monodisperse mesoporous carbon spheres (MCS) were synthesized and their potential applications in ethylene propylene diene monomer (EPDM) foam were evaluated. The obtained MCS exhibited a high specific surface area ranging from 621-to 735 m^2^/g along with large pore sizes. It was observed that the incorporation of MCS into EPDM foam rubber significantly enhances its mechanical properties. The prepared MCS-40 rubber composites exhibit the highest tear strength of 210 N/m and tensile strength of 132.72 kPa, surpassing those of other samples. The enhancement mechanism was further investigated by employing computer simulation technology. The pores within the MCS allowed for the infiltration of EPDM molecular chains, thereby strengthening the interaction forces between the filler and matrix. Moreover, a higher specific surface area resulted in greater adsorption of molecular chains onto the surface of these carbon spheres. This research offers novel insights for understanding the enhancement mechanism of monodisperse mesoporous particles/polymer composites (MCS/EPDM) and highlights their potential application in high-performance rubber composites.

## 1. Introduction

Rubber materials are extensively employed in both industrial and everyday applications. Ethylene propylene diene monomer (EPDM) rubber, a prevalent type of synthetic rubber, exhibits excellent properties that rely on its internal structure and the additives used, making it increasingly utilized in various technical and industrial applications [1]. The selection of fillers generally impacts both the physical properties and the production costs. Commonly used fillers include carbon black [2], silica [3], calcium carbonate [4], and kaolin [5]. The use of innovative fillers to achieve high-performance rubber offers extensive prospects. The behavior of fillers in elastomer matrices is heavily influenced by the filler’s structure (specific surface area, shape, functional groups), the characteristics of the polymer medium (polarity, structure), and the crosslinking system. Recent studies have increasingly focused on mesoporous materials due to their distinctive attributes such as high surface area, large pore volume, and controllable structure [6]. Mesoporous carbon materials, a specific of carbon materials characterized by pore sizes ranging from 2 to 50 nm, are typically synthesized using precursors like rice husk [7], asphalt [8], and phenol-formaldehyde resin [9], followed by physical or chemical activation. Compared to other porous carbon materials such as activated carbon and molecular sieves, mesoporous carbon has gained significant attention in various fields including catalysis, adsorption, gas sensing, and energy conversion and storage due to its exceptional thermal stability, chemical inertness, and excellent conductivity. However, the potential of mesoporous carbon materials in the rubber sector, particularly in EPDM foam materials, remains largely unexplored. The discovery of fullerenes and carbon nanotubes has sparked considerable interest in the exploration of various shaped carbon materials, such as fibers [10], spheres [11], horns [12], and flasks [13]. Extensively studied for decades, spherical carbon materials have found wide-ranging applications in chromatography [14], as catalyst supports, and in rubber reinforcement [15]. Kseniia et al. have reported that the geometric shape of carbon fillers significantly influences elastomer properties [16]. The enhancement effect of carbon materials is influenced by their shape and interaction, which determines their interaction and distribution within the matrix. Carbon materials with different shapes, such as nanodiamonds [17], graphene nanoplates [18], and multi-walled carbon nanotubes [19], exhibit distinct dispersion and orientation states in elastomer composites due to their unique geometric features, thereby affecting mechanical properties including strength, toughness, and stiffness. For example, layered graphene nanoplates can form more effective mechanical reinforcement networks within materials, while nanotubes may provide superior bending strength and toughness. Spherical carbon materials [15] promote better dispersion.

The primary approach for synthesizing mesoporous carbon materials is template casting [20], which involves replicating the internal skeletal structure of various mesoporous inorganic templates in carbon materials. To date, different structures of mesoporous carbon nanospheres have been successfully synthesized using methods such as soft templating [21] and hard templating [22]. For example, Tang et al. reported a soft templating method that involved blending diblock copolymer PEO-b-PS with polydopamine to prepare mesoporous carbon nanospheres [23]. Recently, Wang et al. synthesized mesoporous carbon nanospheres by using polyaniline as a nitrogen source and colloidal silica as a hard template [24]. However, this nanocasting strategy is laborious, intricate, and time-consuming. In recent years, hydrothermal carbonization (HTC) of carbohydrates has gained recognition as an environmentally friendly and sustainable process for synthesizing spherical carbon-based materials [25]. The main objective in preparing carbon sphere materials lies in ensuring their monodispersity while being able to control their outer dimensions and chemical properties [11]. Nevertheless, during practical synthesis, hydrothermally synthesized carbon spheres from sucrose as the carbon source often exhibit interconnections and irregularities. Therefore, achieving simple preparation of monodisperse mesoporous carbon spheres holds significant importance.

Rubber composite materials consist of polymers with molecular weights ranging from several hundred to several thousand and fillers with micro- and nano-scale diameters. A range of EPDM rubber composites containing carbon nanoparticles have been synthesized. Eyssa et al. [26] fabricated sponge ethylene propylene diene rubber (EPDM) nanocomposites based on functionalized multi-walled carbon nanotubes (f-MWCNTs) and foaming agent azodicarbonamide (AZD), which show significantly improved mechanical properties. Shojaei Dindarloo et al. [27] report the effect of various nano-particle types and concentrations on vulcanization and mechanical characteristics of EPDM rubber foam, which demonstrates that the foam’s properties were efficiently influenced by both the shapes and content of the nanoparticles in the matrix. Utilizing appropriate nanoparticles is highly advantageous for enhancing the mechanical properties of foamed rubber. Monodispersed mesoporous carbon spheres, due to their uniform size distribution and mesoporous structure, offer significant advantages over other nanoparticles as fillers in EPDM rubber foams. Their unique structural characteristics may lead to more effective stress distribution and enhanced mechanical properties, thereby presenting a novel and potentially more efficient method for improving the performance of EPDM rubber foams. However, due to the significant size difference between the fillers and polymer chains, accurate calculation of the mechanical properties using full atomistic molecular dynamics simulations is not feasible [28]. To overcome this limitation, coarse-grained molecular dynamics simulations were conducted utilizing the Kremer–Grest chain (bead-spring model). In such simulations, each bead in a chain represents multiple monomer units within a molecular model [29]. Although the chemical effects resulting from chain linking are not considered in coarse-grained molecular dynamics simulations, appropriate potential energies between beads (such as bond and nonbond potentials) enable the simulation of chain dynamics at the micro-scale level.

The present study focuses on the synthesis of monodisperse mesoporous carbon spheres and explores their potential application in EPDM rubber foam. To achieve this, uniform-sized and mesoporous carbon spheres were successfully synthesized using a polymer dispersant method. These carbon spheres exhibited high specific surface areas (621–735 m^2^/g) and large pore sizes, with adjustable structural features by controlling the amount of sodium polyacrylate. The impact of incorporating these mesoporous carbon spheres as fillers into EPDM foam rubber on mechanical properties was examined. Due to the unique properties of the mesoporous carbon spheres, better integration with the rubber matrix was achieved, resulting in enhanced strength and hardness of the EPDM foam rubber. Furthermore, a coarse-grained molecular dynamics model was established to simulate the stress–strain curve of rubber composite materials under tension, providing insights into the mechanism behind this enhancement effect. This research offers valuable insights for understanding the enhancement mechanism of monodisperse mesoporous particles/polymer composites and highlights their potential application in high-performance rubber composites.

## 2. Materials and Methods

### 2.1. Chemicals

Ludox HS-40 (40 wt%, 12 nm), sucrose (C_12_H_22_O_11_, 99.5%), and poly (acrylic acid sodium salt) were provided by Sigma Aldrich (St. Louis, MO, USA). EPDM was obtained from the Jilin Chemical Industrial Limited Company of China (Jilin, China). Carbon black N550 was obtained from Cabot Corporation (St. Boston, MA, USA) in the United States; foaming agent AC was acquired from Changzhou Yongxin Fine Chemical Co., Ltd. (Changzhou, China). The antioxidant 2246, zinc oxide (ZnO), stearic acid (SA), accelerator N-cyclo-hexylbenzothiazole-2-sulphenamide (CZ), sulfuric acid (H_2_SO_4_, 98%), sodium hydroxide (NaOH), and insoluble sulfur (S) are all industrial grade products from Kemiou Chemical Co., Ltd. (Tianjin, China).

### 2.2. Synthesis of Monodisperse Mesoporous Carbon Sphere

Sucrose was the precursor utilized in this study. Commercial silica sol (Ludox HS-40) was used as the templating agent, maintaining a sucrose/silica dioxide molar ratio of 5.5:1. An amount of 50 mL of 0.66 M sucrose solution requires the addition of 7 mL of Ludox HS-40, concentrated 1.25 mL sulfuric acid was introduced as a catalyst. Continuously stir the mixture for 30 min to ensure the formation of a homogeneous solution. Subsequently, the solution was then pre-carbonized by heating at 100 °C for 24 h. The resulting sludge-like pre-carbonized product was separated and washed with deionized water until neutral pH was achieved. The sludge-like resultant mixture was dried at 100 °C for 6 h and a dark brown powder was obtained. Following that, the product was calcined at 900 °C for 3 h under an inert atmosphere (N_2_). Finally, the silica template was removed by NaOH solution (2M) at room temperature, with stir overnight. The mesoporous carbon spheres (MCS) were then washed with ethanol and deionized water.

To prepare monodisperse mesoporous carbon spheres, sodium polyacrylate (0.04% by mass) was added prior to adding the templating agent, followed by stirring for 8 h to ensure complete dissolution before rapidly introducing the templating agent and catalyst into the mixture. The remaining steps were identical to those for the synthesis of MCS. The resulting product is referred to as MCS-X (where X represents the amount of sodium polyacrylate added).

### 2.3. Synthesis of MCS/EPDM Rubber Composites

MCS/EPDM rubber composites were synthesized via two-roll open mill. First, EPDM (100 phr) was masticated for 2 min using an open two-roll mill (ZG-200L, Dongguan Zhenggong Mechanical and Electrical Equipment Technology Co., Ltd., Dongguan, China), Then, zinc oxide (5 phr), stearic acid (1 phr), carbon black N550 (8 phr), MCS (1phr), antioxidant 2246 (2 phr), accelerator CZ (1 phr), foaming agent AC (4phr), and sulfur (1.6 phr) were sequentially added to the masticated EPDM over a period of 30 min until a homogeneous mixture was formed [30]. Finally, the material was cooled to room temperature and then subjected to foaming in an oven at a heating rate of 0.5 °C/min up to 160 °C.

### 2.4. Characterization and Performance Tests

#### 2.4.1. Characterization

The textural properties of MCS were evaluated through nitrogen adsorption–desorption isotherms at −196 °C, using a Micromeritics ASAP-2460 adsorption apparatus(Micromeritics Co., Ltd., St. Norcross, GA, USA). Both specific surface area and pore volume were determined using the BET (Brunauer–Emmett–Teller) equation and the single-point method, respectively. Pore size distribution (PSD) curves were evaluated by BJH (Barrett–Joyner–Halenda) method. Furthermore, the t-plot method was employed to determine the micropore volume and mesoporous surface. Scanning electron microscopy (SEM) images were captured by an electron microscope (Hitachi Ltd., Tokyo, Japan, SU8010) at an electron beam voltage of 1.0 kV. The particle size statistics and measurements on the SEM images of the MCS were analyzed using Nano Measurer 1.2.5.

#### 2.4.2. Testing of the MCS/EPDM Rubber Composites

The mechanical properties of MCS/EPDM rubber composites were determined in accordance with the national testing standards of China (GB/T 6344-2008 [31], GB/T 10808-2006 [32], and GB/T 6669-2008 [33]), using a Universal Materials Testing Machine (model CMT6102, Shenzhen Sansi Experimental Equipment Co., Ltd., Shenzhen, City).

Tensile strength and elongation at break are crucial performance parameters for foamed rubber products. The MCS/EPDM rubber composites were made into 13 mm × 152 mm dumbbell-shaped samples with a gauge length of 50 mm. Sample thickness was measured by dial calipers. The universal material testing machine employed in this study operated at a speed of 500 mm/min. A total of ten samples were tested under each loading condition.

Tear strength measures the material’s resistance to tearing and it is related to the durability of the foam. The MCS/EPDM rubber composites were made into 25 mm × 25 mm × 125 mm rectangular samples with a 50 mm center cut along. The two resulting strips from the razor notch are pulled apart using the universal material testing machine (Shenzhen Sansi Experimental Equipment Co., Ltd., Shenzhen, China) at a rate of 10 mm/min until a minimum of 20 mm length is torn. The maximum force is then recorded and divided by the sample thickness. Five samples were tested under each loading condition.

The compression strength test measures the load-bearing capacity of the foam after specified conditions of time and temperature. The MCS/EPDM rubber composites were made into 50 mm × 25 mm (diameter × height) samples. The compression ratio was 50%. The speed of the universal material testing machine was 2 mm/min. Three samples were tested under each loading condition.

#### 2.4.3. Coarse-Grained Molecular Dynamics Simulation

The elongation behavior of MCS/EPDM rubber composites was simulated by using the simple bead–spring model. Nonbonded interactions are governed by a Lennard–Jones potential energy function of Formulation (1) [34].

(1) $$U_{\mathrm{LJ}\left(r\right)=}\left\{\begin{array}{c} 4\varepsilon \left[{\left(\frac{\sigma }{r}\right)}^{12}-{\left(\frac{\sigma }{r}\right)}^{6}\right],\;\;\;\;\;\;\;\;\;\;\;\;\;\;\;\;\;\;\;\;\;r\leq r_{in}\\ \sum_{j=0}^{4}C_{j}{\left(r-r_{in}\right)}^{j},{\;\;\;\;\;\;\;\;\;\;\;\;\;\;\;\;\;\;\;\;r}_{in}<r\leq r_{c}\\ 0,\;\;\;\;\;\;\;\;\;\;\;\;\;\;\;\;\;\;\;\;\;\;\;\;\;\;\;\;\;\;\;\;\;\;\;\;\;\;\;\;\;\;\;\;\;\;\;\;\;\;\;\;r>r_{c} \end{array}\right.$$

where *ε* and *σ* are the Lennard–Jones parameters for energy and length, respectively, is the distance between any two beads, *r_c_* is a cutoff distance, *r_in_* is the inner cutoff distance for a smoothed potential, and the C values are constants calculated so that the force and its first derivative go smoothly from the Lennard–Jones potential at the inner cutoff to zero at the outer cutoff distance.

The value of *ε* is unity for polymer–polymer and particle–particle interactions, thereby promoting mixing or dispersion of the rods within the polymer matrix. The bonded energy between two consecutive beads in the same chain/particle is given by a harmonic function of the form.

(2) $$U_{bond}\left(r\right)=k_{r}{\left(r-r_{0}\right)}^{2}$$

The rodlike character of the inclusions is enforced through a bending potential of the form.

(3) $$U_{bend}\left(r\right)=k_{\theta }{\left(\theta -\theta_{0}\right)}^{2}$$

In this simulation calculations, where the spring constant is set to *k_r_* = 10^3^ and 10^4^ ×*ε*/*σ*^2^ for polymer bonds and nanorod bonds, *k_θ_* is set to 200 *ε* rad^−2^. In the coarse-grained model, the box size was set to 50^3^, containing 20 MCS units, and a volume fraction of 0.25%. This model was used to simulate and analyze the impact of the filler’s specific surface area on the mechanical properties of rubber composites.

## 3. Results

### 3.1. Characterization of Mesoporous Carbon Spheres

During the synthesis process, sodium polyacrylate (PAANa) plays a pivotal role in regulating the uniformity of the synthesized mesoporous carbon spheres. Gong et al. [35] have proposed that incorporating a dispersant in the hydrothermal synthesis of carbon spheres facilitates the formation of uniformly sized carbon spheres. To investigate the influence of the dispersants on the synthesis of mesoporous carbon spheres, Ludox HS-40 was selected as the template. After the addition of sodium polyacrylate during the synthesis process, the resulting product was designated as MCS-X, where X represents the quantity of sodium polyacrylate added. This study aims to elucidate how sodium polyacrylate impacts and enhances the uniformity of mesoporous carbon spheres.

**Figure 1:**
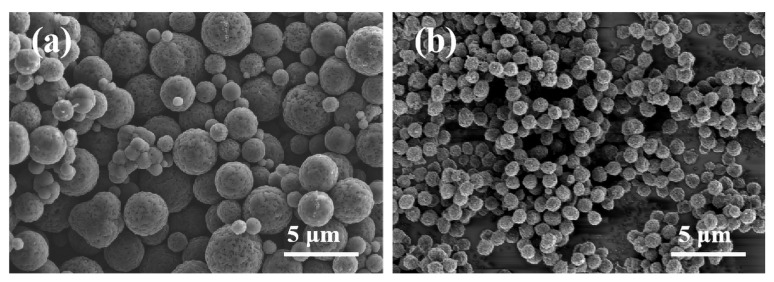
SEM images of mesoporous carbon spheres MCS (**a**) and MCS-40 (**b**).

**Figure 2:**
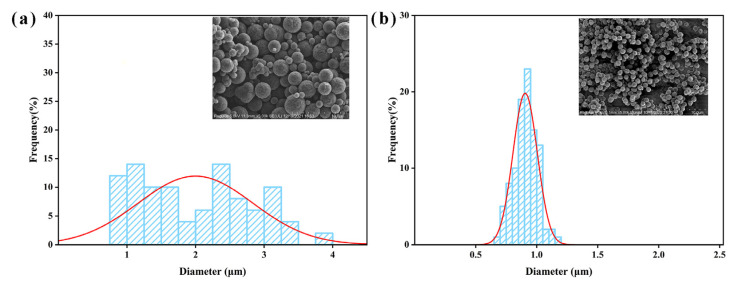
Particle size distribution histograms of (**a**) MCS and (**b**) MCS-40.

Figure 1 illustrates the SEM images of MCS and MCS-40. As shown in Figure 1a, the mesoporous carbon spheres synthesized without the dispersant resulted in a spherical morphology. However, there was noticeable heterogeneity in particle size, accompanied by some degree of adhesion and aggregation. This phenomenon can be attributed to the high temperatures during synthesis, which induced nucleation and subsequent condensation of sucrose molecules, ultimately leading to irregular and crosslinked carbon spheres [36]. In contrast, as shown in Figure 1b, the addition of PAAN facilitated the formation of monodisperse and uniform carbon spheres [37]. The prepared spheres exhibited complete monodispersity with a consistent size (0.9–1.0 μm), and no other shapes of particles or fragments were observed within the samples. Particle size distribution analysis of both types of mesoporous carbon spheres is performed. Figure 2a demonstrates that MCS exhibits a broad range of particle sizes, varying from 0.9 to 4.0 μm, indicating an uneven size distribution within the material. In contrast, Figure 2b illustrates that MCS-40 displays a narrow particle size distribution, centered around 0.9–1.0 μm, suggesting a high level of uniformity in terms of particle size. This narrow distribution signifies the successful synthesis of monodisperse mesoporous carbon spheres.

**Figure 3:**
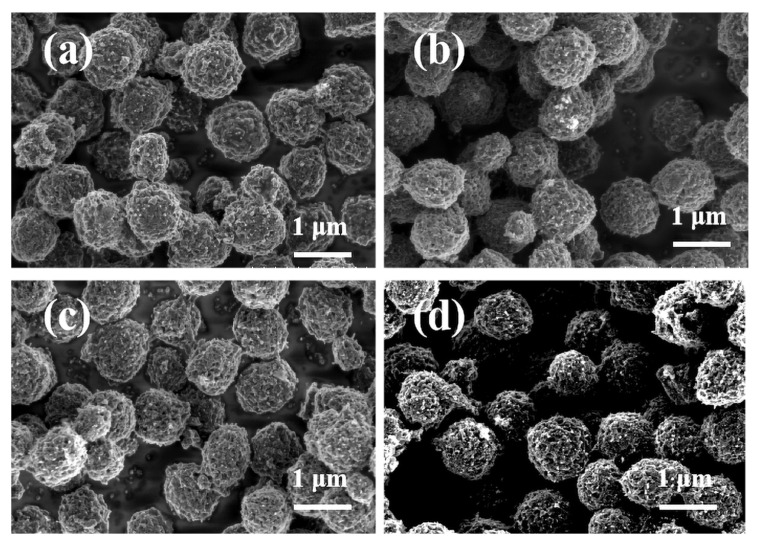
SEM images of mesoporous carbon spheres MCS-X (X is the amount of sodium polyacrylate added in mg). (**a**) MCS-20; (**b**) MCS-25; (**c**) MCS-30; and (**d**) MCS-40.

To investigate the impact of sodium polyacrylate (PAANa) on the morphology of synthesized mesoporous carbon spheres, varying amounts of PAANa were utilized during the synthesis process. Figure 3 demonstrates a significant enhancement in the uniformity of mesoporous carbon spheres upon the addition of this dispersant, resulting in an overall more uniform particle size. However, with increasing amounts of PAANa, agglomeration among the mesoporous carbon spheres was observed. Specifically, when 40 mg of dispersant was added, some carbon spheres showed slight fragmentation, which affected their spherical morphology. This phenomenon can be attributed to excessive aggregation of excessive dispersant that impedes proper encapsulation and carbonization processes for the carbon source, ultimately leading to surface damage in the formed mesoporous carbon spheres [35].

### 3.2. Morphology of the Foams

**Figure 4:**
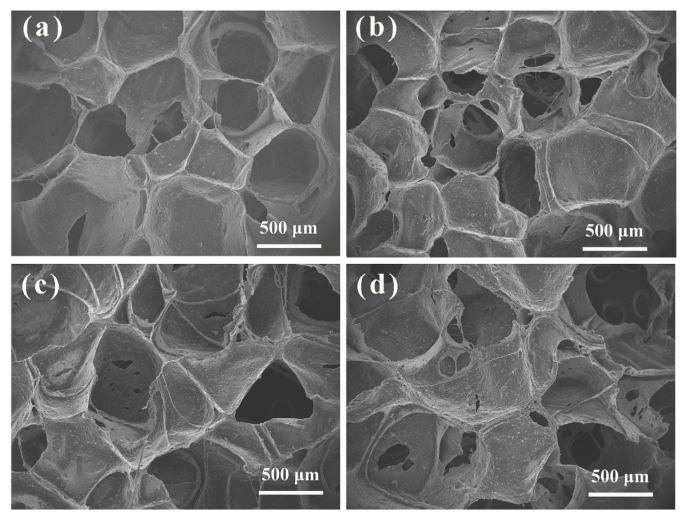
SEM images of the MCS/EPDM nanocomposite foam samples containing (**a**) MCS-20, (**b**) MCS-25, (**c**) MCS-30, and (**d**) MCS-40.

The SEM images of the foam samples are shown in Figure 4 which revealed the presence of both open cells and closed cells within the porous structure of the foams. The white parts represent closed pores and the dark part represents open pores. The semi-open cell structure represented the edges and surfaces along with the existence of some holes in the cell walls. These pores allow gas molecules to pass through the continuous phase, thereby creating fluidity through the foam to some extent [35]. Furthermore, nano-fillers acted as active nucleating agents, providing more bubble sites. The effect of MCS on the pore structure of EPDM foams was not significant. The effect of MCS on the mechanical properties of EPDM foams is mainly due to the interaction between MCS and rubber matrix.

### 3.3. Specific Surface Area and Pore Size Analysis of Mesoporous Carbon Spheres

**Figure 5:**
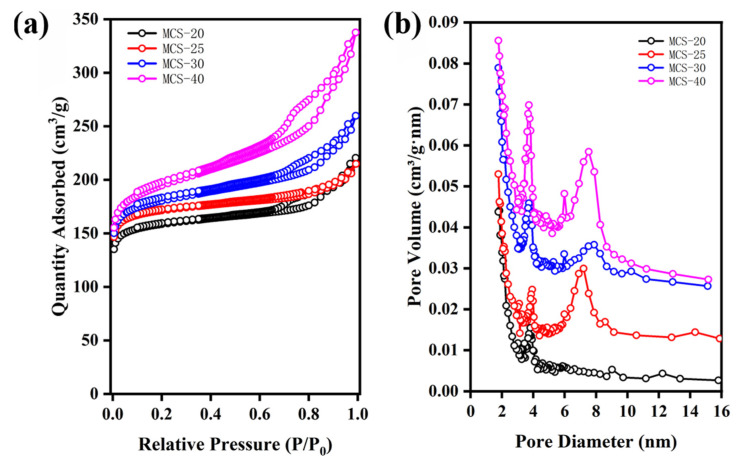
N_2_ adsorption and desorption curves of MCS with different contents of sodium polyacrylate: (**a**) N_2_ adsorption and desorption curves and (**b**) pore size distribution.

Table 1 presents the characterization results of mesoporous carbon spheres (MCS) synthesized with varying weights of sodium polyacrylate (PAANa). Figure 5 shows the nitrogen adsorption–desorption isotherms and the corresponding pore size distribution curves. As depicted in Figure 5a, MCS-X exhibits Type IV BET adsorption isotherms according to IUPAC classification, accompanied by H-2 hysteresis loops, which are typical characteristics of mesoporous materials. Furthermore, the hysteresis loop at high pressure (P/P0 = 0.89–0.98) reflects the existence of large mesopores, which could be attributed to the inter-particle packing between the mesoporous carbon nanospheres [38]. Notably, as shown in Figure 5a, both the overall specific surface area and the low-pressure range micropore area increase with the increasing dosage of dispersant PAANa. The microporous specific surface area was determined using the t-plot method, which involves calculating the microporous specific surface area by fitting a linear curve to the adsorption isotherm’s low-pressure region in the microporous adsorption region. For calculating the external surface area (St-plot) from the slope of the linear fit, the relation proposed by Harkins and Jura [39] was employed as the standard reference t-curve, combined with the data presented in Table 1. By analyzing the textural properties of mesoporous carbon spheres with added PAANa, it is evident that the Brunauer–Emmett–Teller (BET)-specific surface areas of the mesoporous carbon spheres are, respectively, 621 m^2^/g, 673 m^2^/g, 695 m^2^/g, and 735 m^2^/g, demonstrating a consistent upward trend. The mesopore size distribution curves derived from the Barrett-–Joyner–Hallenda (BJH) method [40] show that the MCS-40 mesopore diameters are 3 nm and 8 nm, respectively. The micropore-specific surface areas are measured as 631 m^2^/g, 582 m^2^/g, 563 m^2^/g, and 529 m^2^/g. Furthermore, the pore volume exhibits an increase upon the addition of sodium polyacrylate.

### 3.4. Mechanical Properties

**Figure 6:**
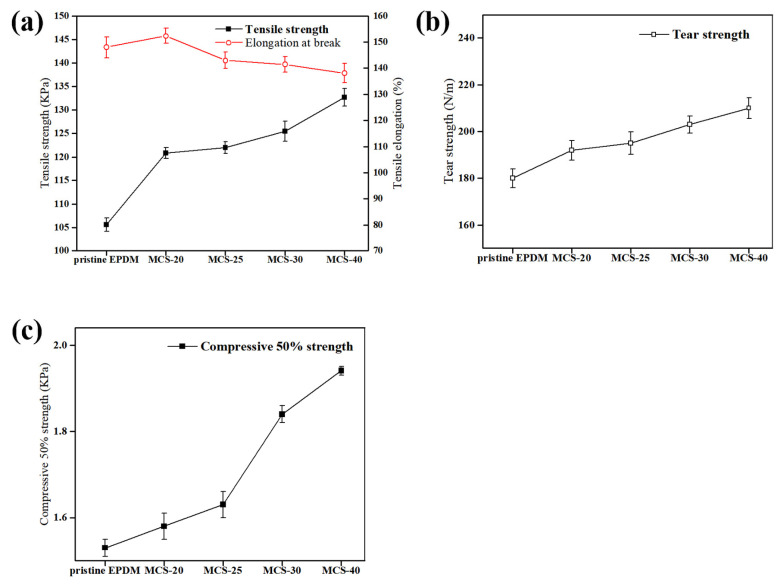
The effect of MCS-X on the mechanical properties of MCS/EPDM rubber composites: (**a**) tensile strength and elongation at break. Values are means ± s.e.m.; n = 10; (**b**) tear strength. Values are means ± s.e.m.; n = 5; (**c**) compress 50% strength. Values are means ± s.e.m.; n = 3.

The impact of different types of mesoporous carbon spheres (MCS) on the mechanical properties of MCS/EPDM rubber composites is shown in Figure 6, while specific values are provided in Table 2. The mechanical properties of EPDM foam materials are improved with the addition of MCS. Figure 6a demonstrates the elongation at break and the tensile strength of EPDM foam materials upon the addition of MCS-X. As shown in Figure 6a, MCS-40 exhibits superior tensile strength, albeit with slightly compromised elongation at break performance. The favorable compatibility between the carbon material and the polymer matrix facilitates effective stress transfer, thereby enhancing the mechanical properties of the composite.

In Figure 6b, it can be observed that MCS-40 possesses the highest tear strength due to the effective restriction of rubber chain segmental motion within the mesoporous channels [41]. Furthermore, as depicted in Figure 6c, MCS-40 displays the best compression strength, which is attributed to the large specific surface area of MCS-40, to allow for better bonding with the rubber matrix. The addition of PAANa results in a larger specific surface area of MCS, which promotes increased binding glue formation leading to enhanced reinforcement, elevated tensile strength, and greater load-bearing capacity.

In the coarse-grained model, the forces utilized include the harmonic potential and the Lennard–Jones potential. The harmonic potential is employed to simulate bonded interactions, while the Lennard–Jones potential represents non-bonded interactions. These forces play a crucial role in simulating the behavior and properties of filled cross-linked rubber [35]. Moreover, within this coarse-grained model, variations in filler-specific surface area directly impact the non-bonded interactions, whereby larger surface areas correspond to stronger forces [42].

**Figure 7:**
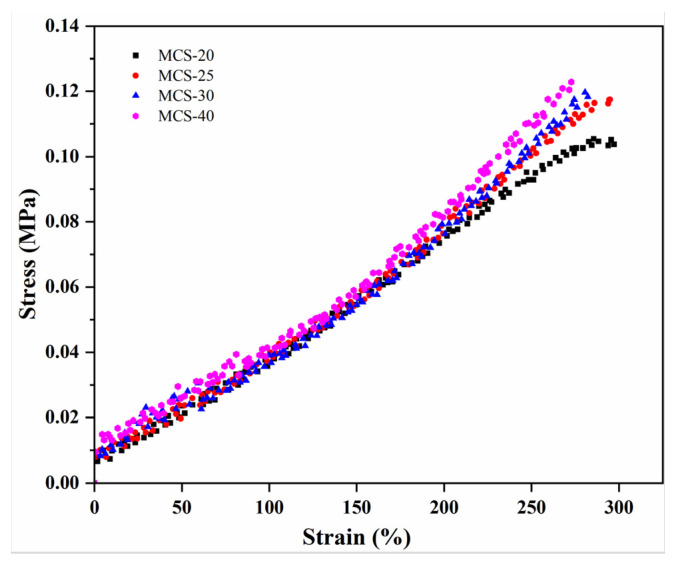
Simulated stress–strain elongation curves in the uniaxial of MCS/EPDM rubber composites.

Figure 7 shows the stress–strain curves in the uniaxial elongation simulation of the filled crosslinked polymer model. During the elongation process, the stress was influenced by the strength of the filler–polymer interaction, with a stronger interaction leading to higher stress. The strongly attractive filler–polymer interaction caused a shift in the stress upturn point towards lower elongation values. Moreover, materials with larger specific surface areas provided more binding sites, thereby enhancing the interaction forces between the filler and rubber [43]. As shown in Figure 7, MCS-40 with a larger specific surface area exhibits greater stress and a lower elongation rate compared to other samples under identical conditions.

**Figure 8:**
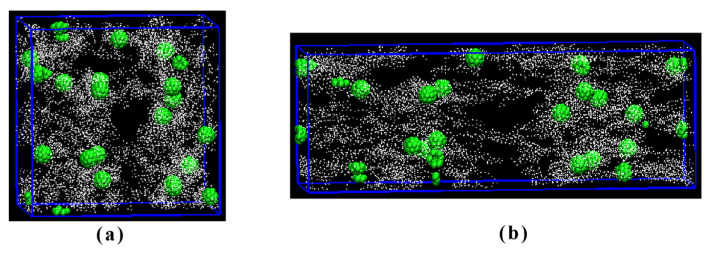
Coarse-grained models of the composites with MCS-40, (**a**) 0%, and (**b**) 200% elongation.

Figure 8 shows the images of the coarse-grained model at 0% and 200% stretch, where the green balls represent the synthetic monodisperse mesoporous carbon spheres. Surrounding these carbon spheres are white polymer chains of EPDM, simulating the adsorption of the EPDM molecular chains onto the surface of carbon spheres. The pore structure on the carbon sphere’s surface facilitates enhanced interaction forces by allowing penetration of EPDM molecular chains into these pores. Consequently, a larger specific surface area leads to the increased adsorption of molecular chains onto the surface of this carbon ball. Adding the filler resulted in a non-linear viscoelastic behavior, known as the Payne effect. The higher the surface area the higher the Payne effect, which corresponds to the extent of filler networking. The influence of the mesoporous carbon sphere surface area on the formation of a filler network can be explained as follows: At fixed levels of structure and filler loading, both the aggregate size and the inter-aggregate distance decrease with, respectively, increasing surface area [44]. The smaller the inter-aggregate distance the higher the probability for the formation of a filler network. Consequently, when the surface area increases, the extent of the packing network is more pronounced, which is manifested as an increase in tensile strength. During the stretching process, the surface of the molecular chain is also stretched; thus, stronger surface forces result in higher tensile strength while weaker ones lead to lower tensile strength. At 200% stretching, both the orientation of molecular chains and motion-induced changes in the mesoporous carbon sphere’s molecular weight align with the direction of applied tensile force.

## 4. Conclusions

In summary, new monodisperse mesoporous particles/polymer composites (MCS/EPDM) were successfully synthesized by incorporating monodisperse mesoporous carbon spheres (MCS) into ethylene propylene diene monomer (EPDM) foam. The synthesized MCS has a uniform size and mesoporous structure, as well as a high specific surface area. The infiltration of EPDM molecular chains within the pores of MCS strengthened the interaction forces between the filler and matrix. The enhancement mechanism was further investigated by employing computer simulation technology. The simulation of the tensile situation based on coarse-grained molecular dynamics revealed that a higher specific surface area facilitates enhanced accessibility of polymer molecular into the pores of the mesoporous carbon spheres, thus leading to a stronger interaction force between the mesoporous carbon spheres and the polymer chains, ultimately resulting in an enhancement in the tensile strength. This research provides new insights into understanding the enhancement mechanism of MCS/EPDM composites and highlights their potential application in high-performance rubber composites.

## Figures and Tables

**Table 1:** Textural properties of mesoporous carbon spheres added with PAANa.

	S_BET_^a^ (m^2^/g)	S_t-plot_^b^ (m^2^/g)	V_p_^c^ (cm^3^/g)	Pore Size (nm)
MCS-20	621	631	0.33	3.0; 7.0
MCS-25	673	582	0.25	3.0; 9.0
MCS-30	695	563	0.40	3.0; 8.0
MCS-40	735	529	0.52	3.0; 8.0

^a^ Specific surface area was calculated by BET method based on nitrogen adsorption isotherm. ^b^ Specific surface area of micropores calculated by the t-plot method. ^c^ Total pore volume calculated from nitrogen adsorption data with P/P0 = 0.99.

**Table 2:** Mechanical properties of MCS/EPDM rubber composites.

	Filler Type				
	Pristine EPDM	MCS-20	MCS-25	MCS-30	MCS-40
Tensile strength (KPa)	105.66 (±1.42)	120.88 (±1.17)	122.00 (±1.24)	125.45 (±2.15)	132.72 (±1.83)
Elongation at break (%)	148.00 (±4.07)	152.43 (±2.99)	143.00 (±3.08)	141.45 (±2.97)	138.15 (±3.62)
Tear strength (N/m)	180 (±4.12)	192 (±4.17)	195 (±4.76)	203 (±3.63)	210 (±4.5)
Compressive 50% strength (KPa)	1.53 (±0.02)	1.58 (±0.03)	1.63 (±0.03)	1.84 (±0.02)	1.94 (±0.01)

## Data Availability

The data that support the findings of this study are available from the first author, Tong Zheng, upon reasonable request (due to privacy).
